# Influence of Diabetes on Implant Failure and Peri-Implant Diseases: A Retrospective Study

**DOI:** 10.3390/dj8030070

**Published:** 2020-07-04

**Authors:** Alice Alberti, Paolo Morandi, Beatrice Zotti, Francesco Tironi, Luca Francetti, Silvio Taschieri, Stefano Corbella

**Affiliations:** 1 Department of Biomedical, Surgical and Dental Sciences, Universitaà degli Studi di Milano, 20122 Milan, Italy; paolo.morandi@unimi.it (P.M.); zotti.beatrice@gmail.com (B.Z.); francesco.tironi@unimi.it (F.T.); luca.francetti@unimi.it (L.F.); silvio.taschieri@unimi.it (S.T.); stefano.corbella@unimi.it (S.C.); 2 IRCCS Galeazzi Orthopedic Institute, 20161 Milan, Italy; 3 Department of Oral Surgery, Institute of Dentistry, I.M. Sechenov First Moscow State Medical University, 119146 Moscow, Russia

**Keywords:** dental implants, diabetes, hyperglycemia, implant failure, peri-implantitis

## Abstract

Diabetes is an important modifying factor of periodontitis, but its association with peri-implant diseases has not been fully explored and the existing literature reports controversial results. The aim of this retrospective study was to evaluate the influence of diabetes on peri-implantitis and implant failure. Smoking status, history of periodontal disease, presence of diabetes, diabetes type, therapy and glycaemia levels were collected in a total of 204 subjects treated with 929 implants, with a mean follow-up time of 5.7 ± 3.82 years after loading. Odds ratio (OR) for diabetes as a direct cause of peri-implantitis and implant failure were calculated, adjusted for smoking status and history of periodontitis. Nineteen patients were diabetic and most of them presented a good control of the disease at the time of surgery. The overall patient-level prevalence of peri-implantitis was 11.3%. Among diabetic patients, one developed peri-implantitis, whereas one experienced multiple implant failures. The calculated ORs, adjusted for smoking status and periodontitis, were not statistically significant. The results revealed no association between diabetes and peri-implantitis or implant failure coherently with the existing scientific literature. The actual influence of hyperglycemia on implant failure is still uncertain and new studies with larger cohorts of patients are needed.

## 1. Introduction

In the last few decades, the scientific evidence on biofilm-related inflammatory peri-implant diseases has substantially increased. Peri-implantitis, which leads to progressive marginal bone loss around implants, represents the main cause of late implant failure. A new definition has been settled for peri-implantitis in the World Workshop on Periodontology (WWP) in 2017 [1], where the diagnosis of peri-implantitis required: (a) the presence of bleeding and/or suppuration on gentle probing; (b) an increased probing depth compared to previous examinations (in the absence of previous examination: PD ≥ 6 mm); (c) further bone loss as compared to the initial bone remodeling (in the absence of previous examination: bone levels ≥3 mm apical of the most coronal portion of the intraosseous part of the implant). The lack of a univocal definition in the past led to controversial results, with reported prevalence rates ranging from 1.1% to 85% on the implant level [2] and from 0 to 39.7% on the patient level [3]. History of periodontitis and low hygiene levels are proven risk factors for peri-implantitis, while the role of other factors, including smoking status and diabetes, is still unclear. Diabetes mellitus (DM) comprises a group of metabolic disorders characterized by hyperglycemia, which is due to the impairment of insulin secretion and/or action. The incidence of DM and its prevalence has been increasing significantly over the last few decades. The International Diabetes Federation estimated 451 million cases of diabetes in 2017, which represent a global prevalence of 8.4% and are expected to rise [4]. While diabetes has been proven as an important risk factor for periodontitis, its association with peri-implant diseases has not been fully explored, and the existing literature reports controversial results. Some hypotheses of how diabetes could interfere with implant success have been formulated, and these include: the suppression of osteoblastic differentiation, proliferation and activity, deficits in the healing process, and the alteration of the immune response [5].

The primary aim of this retrospective study was to evaluate the relationship between the presence of diabetes and the occurrence of biological complications at the implant site, namely the development of peri-implantitis and of post-operative complications; the secondary aim was to evaluate its association with implant survival rate.

## 2. Materials and Methods

The clinical records of all subjects treated with implants during the period between 1 January 2005, and 31 December 2018 in the Dental Clinic of the IRCCS Istituto Ortopedico Galeazzi (Milan, Italy) were screened. The following inclusion criteria were adopted for clinical record selection: (a) 18-year-old or older patients at the time of intervention; (b) patients who gave their written informed consent for the use of their clinical records for research purposes; (c) patients whose implants present complete clinical and radiographical records, including at least one radiograph per year and a report of complications. Patients lost at follow-ups were excluded from the study.

### 2.1. Outcomes

The primary outcome was the correlation between the presence of diabetes and the development of peri-implantitis. The secondary outcomes were: patient—and implant-level cumulative prevalence of peri-implantitis, patient—and implant-level cumulative implant survival rate, and prevalence of post-operative complications that occurred immediately after the surgical intervention.

### 2.2. Data Collection

The following parameters were collected: gender; age at the time of surgery; ASA score; presence of systemic diseases, smoking status, history of periodontal disease, and presence of diabetes; in case of diabetes, diabetes type, diabetes therapy at surgery, glycaemia levels, and glycated hemoglobin (HbA1c) before surgery, glycosuria and leukocyte formula before surgery were also registered; implant type and characteristics (width, length); prosthesis type (fixed partial dentures, full arch fixed dentures, full arch removable dentures); date of diagnosis of peri-implantitis; date of implant loss/removal. The diagnosis of diabetes was formulated according to the American Diabetes Association guidelines [6]. Peri-implantitis was defined as the presence of bleeding and/or suppuration on gentle probing, together with at least 2 mm bone resorption, evaluated through the comparison of baseline and follow-up periapical radiographs [7]. All the clinical and radiographic records were re-analyzed to verify the diagnosis of peri-implantitis according to the most recent definition [1].

### 2.3. Statistical Analysis

The Shapiro–Wilk tests served to evaluate the normality of the distribution of the variables considered. Descriptive statistics were provided by means of mean values and standard deviations for normally distributed variables.

The cumulative survival rate was calculated by means of survival tables. The absolute patient-level prevalence of peri-implantitis was calculated for diabetic and non-diabetic subjects. Correlation between baseline parameters and outcomes was provided through the use of logistic regression. Odds ratio (OR) for diabetes as a direct cause of peri-implantitis and implant failure were calculated, adjusted for smoking status, history of periodontitis, gender, age, ASA score, presence of systemic diseases, implant type and characteristics, and prosthesis type) on survival curves. The level of significance was *p* < 0.05.

## 3. Results

A total of 204 patients and 929 implants were included. A wide range of implant systems were used, but all of them presented an internal connection with a polygonal design. Among all included subjects, 90 were males and 114 were females, the mean age at the time of surgery was 57.3 ± 13.7 years, 127 had a history of periodontitis, 50 were smokers, and 18 were former smokers. The mean follow-up time was 5.7 ± 3.82 years, varying from 3 months to 15 years after loading. Nineteen patients were diabetic, and most of them demonstrated a good control of the disease. Two subjects presented type 1 DM and were being treated with insulin, while seventeen presented type 2 DM; the details of their therapy is specified in Table 1, together with the descriptive analysis of diabetes-related parameters. Among the diabetic patients, seven received a full-arch implant-supported prosthesis, three received an overdenture prosthesis, six fixed partial dentures, and seven were treated with multiple single crowns; three patients were treated with both fixed partial dentures and single crowns, and one with both an overdenture and a fixed partial prosthesis.

**Figure 1:**
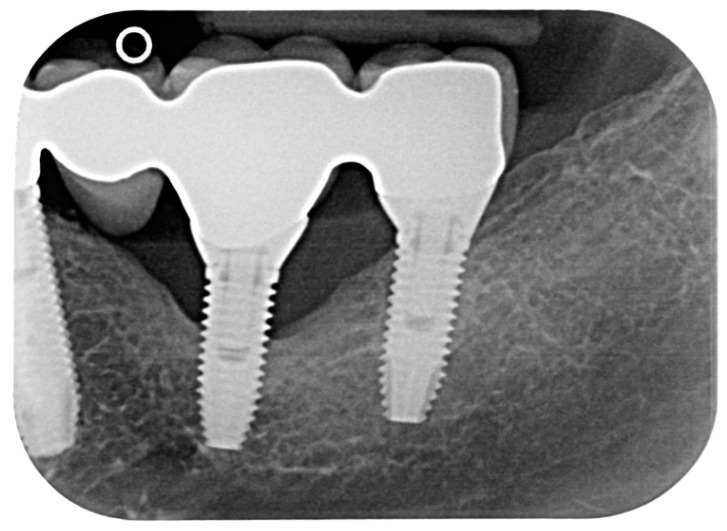
Periapical radiograph of one case of peri-implantitis in a non-diabetic subject.

**Figure 2:**
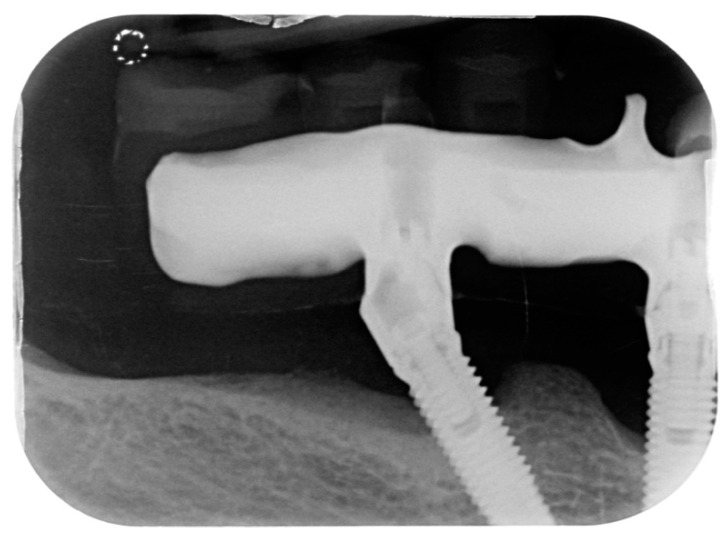
Periapical radiograph belonging to the only diabetic patient who developed peri-implantitis.

**Figure 3:**
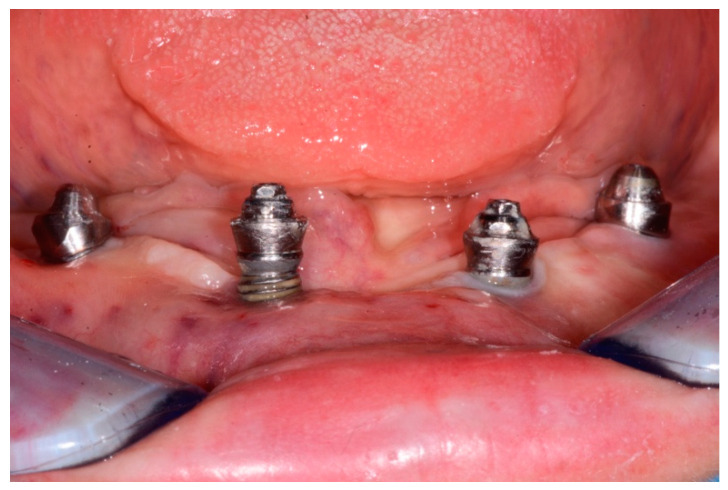
Clinical photograph of the same diabetic patient, showing suppuration and exposure of the implant threads.

A total of 23 cases of peri-implantitis (patient-level) were registered, representing an overall prevalence of 11.3%. Only one diabetic patient (type 2) developed peri-implantitis (5.3%) whereas one subject with type 1 diabetes experienced multiple implant failures due to a failure of osseointegration. Figure 1 and Figure 2 show radiographic evidence of peri-implantitis in one diabetic (Figure 1) and one non-diabetic patient (Figure 2). Figure 1 and Figure 2 show radiographic evidence of peri-implantitis in one non-diabetic (Figure 1) and one diabetic patient (Figure 2). A clinical image of the same diabetic patient is represented in Figure 3.

Patient-level cumulative implant survival rate was 95.42% 10 years after surgery, which was 96.51% and 94.74%, respectively, for diabetic and non-diabetic patients, without any significant difference. None of the diabetic patients experienced post-operative complications.

The OR for diabetes as a cause of peri-implantitis, adjusted for smoking status and history of periodontitis, was not statistically significant (OR = 0.47 (95% C.I. 0.06–3.76)). Similarly, the association between diabetes and implant failure, adjusted for the same proven risk factor, resulted not significative (OR = 1.23 (95% C.I. 0.11–13.30)). The results were adjusted for gender, age, ASA score, presence of systemic diseases, implant type and characteristics, and prosthesis type: none of the above-mentioned parameters were found to influence the development of peri-implantitis.

The small number of cases among diabetic patients prevented us from considering diabetes type and therapy for statistical analysis.

## 4. Discussion

The association between diabetes and the status of peri-implant tissues has been explored extensively in literature, with heterogenous and controversial results [8,9,10]. The results of our study revealed no association between diabetes and the occurrence of peri-implantitis, finding an insignificant OR in the examined cohort. Such results are coherent with those presented by Renvert et al. [11] who did not find a significant OR between a history of type 2 diabetes and peri-implantitis in a cohort of 270 subjects followed-up over time. It must be noted that in the above-mentioned paper, the authors adopted a definition of peri-implantitis that was different from ours, and that could, hypothetically, be the cause of finding a higher prevalence of peri-implantitis. Conversely, Ferreira et al. [12] observed that diabetic patients were more susceptible to develop peri-implantitis, reporting a peri-implantitis prevalence of 24% in diabetic patients and 7% in non-diabetic patients. It must be noted, however, that these results refer to diabetic patients regardless of their glycemic control. In fact, the authors found a higher risk of peri-implantitis, with an adjusted OR of 1.9, for subjects with uncontrolled diabetes, even though the latter was not clearly defined. Daubert et al. [13] also reported a relative risk of 4.1 for peri-implantitis in diabetic patients; however, their study included only five diabetic patients, which could have influenced the statistical analysis in both excess and defect. A meta-analysis published by Monje et al. in 2017 [10] calculated that both the OR and RR (risk ratio) for peri-implantitis were statistically higher in hyperglycemia than in normoglycemia; however, such meta-analysis could not evaluate the impact of smoking and glycemic level because of the lack of information from the included studies.

A relationship between the level of metabolic control of diabetes and peri-implantitis has been suggested in the literature. Venza et al. [14] found that some clinical parameters, including PD and radiographic bone loss, were significantly higher (*p* < 0.05) in poorly-controlled diabetic patients (HBA1c ≥ 8%), as compared to well-controlled diabetic patients (HBA1c < 8%). The authors thus suggested that a poor glycemic control may be involved in the modulation of periodontal destruction and could have a correlation with the severity of peri-implantitis. On the other hand, Gomez-Moreno et al. [15] found that higher HBA1c levels led to higher bone loss over 3 years after implant placement, although this association was not statistically significant. The association between elevated HbA1c levels and increased marginal bone loss had a statistically significant result in a different prospective study by Aguilar-Salvatierra et al. [16]. However, in a recent meta-analysis of seven prospective studies [17], Lagunov et al. observed that PD, BOP, and marginal bone loss showed a significantly higher increase in type 2 DM patients as compared to healthy patients, after a period of 12 months, independently from the level of glycemic control.

Our study only included one poorly-controlled patient, undergoing multiple implant failures. Therefore, no further analysis was possible regarding the development of peri-implantitis. In addition, the patient presented HbA1c ≥ 8.0, but lower than 9%, which is considered as “moderately-controlled diabetes” in some studies. 

Our study did not reveal any statistically significant association between diabetes and post-operative complications, but this could also be due to the small size of the DM group.

As for implant survival rates, the present study did not find any association with diabetes. A recent review published by Oliveira-Neto et al. in 2019 [5] came to the same conclusion, reporting that diabetes did not affect implant survival rate in two meta-analyses of high methodological quality [18,19]. Chrcanovic et al. [18] analyzed a total of 604 subjects (49 diabetic, 555 non-diabetic) and reported an RR of 1.07, while Moraschini and Barboza [19] analyzed a total of 2334 subjects (802 diabetic, 1532 non-diabetic), with an RR of 1.43 and 3.65 for type 1 and type 2 DM, respectively; it must be noted that in all the included studies diabetes was under control at the time of the surgery.

A weakness of the present study is the wide range of follow-up times, which was almost 6 years on average, representing a medium-term follow-up, but reached a minimum of 3 months after functional loading. Such short follow-up time still allows the evaluation of post-operative and short-term complications, but cannot account for a long-term analysis. However, all the diabetic patients but one presented a follow-up of more than 1 year, reaching a maximum of 13 years.

Another limitation of the study is that more than one systemic disease can be found in the same patient, resulting in a confounding factor. It must be noted that the only patient undergoing multiple early failures also had cardiovascular disease.

One of the strengths of our study is the fact that glycemia levels and glycated hemoglobin at surgery were recorded, which could allow the detection of the association between the level of compensation of the disease and the occurrence of early complications. Although the small number of diabetic patients prevented us from performing a specific and “powerful” statistical analysis, the registration and analysis of these parameters are fundamental for further meta-analysis of similar studies. Moreover, it should be noted that the only patient with poor glycemic control (HbA1c ≥ 8.0%) experienced multiple implant failure with a lack of osseointegration. Interestingly, some previous studies reported higher rates of early implant failures in diabetic patients [20,21], and one prospective study by Ghiraldini et al. observed that hyperglycemia negatively affected the implant osseointegration [22]. However, a recent meta-analysis by Shi et al. [23] found no significative association between diabetes and implant failure in patients with both good and poor metabolic control.

A possible limitation of our study could be that glycemia levels and HbA1c were not registered at follow-up visits, preventing us from disclosing a possible association between glycaemia levels in diabetic patients and long-term complications. Even though glycemia levels and HbA1c measured immediately before surgery could be of great relevance in relations to early complications, the subsequent changes in the level of metabolic compensation could be not easily controlled over the years and may influence the onset of long-term biological complications. Hence, further studies analyzing the association between glycemia levels before and after surgery, and the occurrence of biological complications and implant failure, are needed.

It should be underlined that the definition of peri-implantitis proposed by Heitz-Mayfield et al. in 2014 was used in this study, but all the included records were re-analyzed during data collection and the cases of peri-implantitis were confirmed in light of the new definition settled in the WWP in 2017 [1].

## 5. Conclusions

The actual influence of DM and hyperglycemia on peri-implantitis and implant failure is still uncertain and new studies on larger cohorts of patients are needed also taking into consideration further parameters, such as HbA1c baseline and follow-up values, baseline and follow-up diabetic therapy, and duration of diabetes. Future studies are needed to investigate the relationship between the long-term changes in glycemia and HbA1c levels and the health status of peri-implant tissues. Monitoring the main parameters of glycemic control is desirable not only for research purposes, but also for clinicians, since poor metabolic control may lead to complications such as increased risk of infections. Within the limitations of the present study, our results confirm that implant therapy in diabetic patients with good glycemic control should be considered a safe and viable treatment option.

## Figures and Tables

**Table 1:** Clinical parameters of diabetic patients (*n* = 19). Continuous variables are reported as mean ± standard deviation (minimum; maximum). Discrete variables are reported as number of cases.

Parameter	Values Before Surgery
Diabetes type	Type 1: 2 Type 2: 17
Diabetes therapy	Diet only: 5
	Metformin: 7
	Insulin: 3
	Sulfonylureas (glimepiride): 1
	Metformin + sulfonylureas (glimepiride, glibenclamide): 3
	Metformin + pioglitazone + glicazide: 1
Glycemia at surgery (mg/dL)	127.63 ± 25.67 (91; 155)
Glycated haemoglobin at surgery (%)	6.40 ± 0.36 (5.9; 8.0)
Glycosuria (mg/dL)	0 ± 0 (0; 0)
Lymphocytes (10^9^/L)	2.48 ± 0.72 (1.47; 3.43)
Neutrophiles (10^9^/L)	4.19 ± 1.30 (2.40; 6.21)
